# Aichi Virus 1: Environmental Occurrence and Behavior

**DOI:** 10.3390/pathogens4020256

**Published:** 2015-05-19

**Authors:** Masaaki Kitajima, Charles P. Gerba

**Affiliations:** Department of Soil, Water and Environmental Science, The University of Arizona, 1117 E. Lowell St., Tucson, AZ 85721, USA; E-Mail: gerba@ag.arizona.edu

**Keywords:** Aichivirus, kobuvirus, qPCR, occurrence, wastewater treatment, surface waters

## Abstract

Aichi virus 1 (AiV-1), belonging to the genus *Kobuvirus* in the family *Picornaviridae*, has been proposed as a causative agent of human gastroenteritis potentially transmitted by fecal-oral routes through contaminated food or water. AiV-1 is globally distributed and has been detected in various types of environmental samples, such as sewage, river water, groundwater, and shellfish. Recent environmental studies revealed that this virus could be detected in higher frequency and greater abundance than other human enteric viruses. These findings suggest that AiV-1 could potentially be an appropriate indicator of viral contamination in the environment because of its high prevalence in water environments as well as structural and genetic similarity with some of the other important enteric viruses. Further studies on the occurrence and fate of AiV-1 in environments, even in combination with clinical studies of many regions, are needed for a better understanding of their epidemiology, temporal and geographical distribution, environmental stability, and potential health risks to humans.

## 1. Introduction

**Figure 1:**
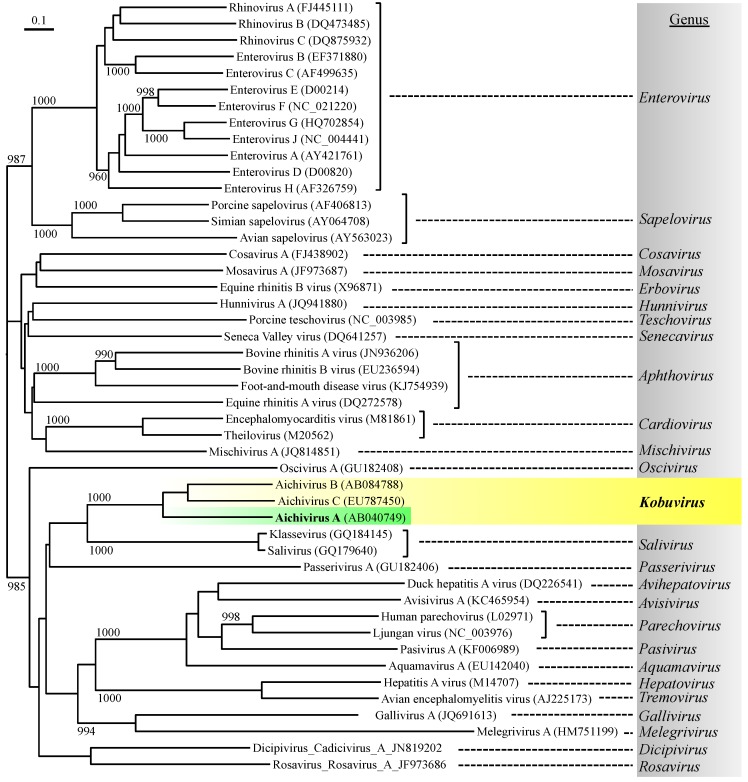
Phylogenetic relationship of Aichi virus 1 with representative species from each picornavirus genus, based on amino acid similarity of the P1 (viral structural proteins) region using the neighbor-joining method. The numbers on each branch indicate the bootstrap values obtained from a bootstrap analysis with 1000 replicates, and the scale represents amino acid substitutions per site.

The family *Picornaviridae* currently consists of 26 recognized genera (International Committee on Taxonomy of Viruses, ICTV; http://www.ictvonline.org/) and contains important human enteric viruses that can cause waterborne infections to humans, such as enteroviruses and hepatitis A virus (Figure 1) [1]. Aichi virus 1 (AiV-1), a human enteric virus belonging to the genus *Kobuvirus*, is also a member of the family *Picornaviridae* [2]. The genus *Kobuvirus*, which is a newly recognized genus, consists of three species: Aichivirus A, Aichivirus B, and Aichivirus C, which were recently renamed and formerly called Aichi virus/Aichivirus, bovine kobuvirus, and porcine kobuviruses, respectively [3]. The species Aichivirus A consists of three genetically distinct members with different host species, namely, AiV-1 (Aichivirus in humans) [4], canine kobuvirus 1 [5], and murine kobuvirus 1 [6].

**Figure 2:**
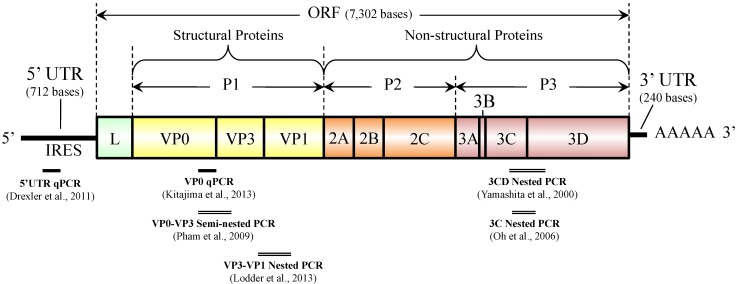
Genome organization of Aichi virus 1 and target locations of diagnostic PCR assays.

AiV-1 possesses a single-stranded, positive-sense RNA genome approximately 8.3 kb in length, which is comprised of a 5' untranslated region (UTR) with an internal ribosomal entry site (IRES), a large open reading frame (ORF) of approximately 7.3 kb encoding a single polyprotein (putative protein precursor), and a 3' UTR region [2,7,8,9]. As shown in Figure 2, a non-structural leader (L) protein is encoded at the N-terminus of the polyprotein, followed by viral structural proteins P1 (VP0, VP3, and VP1) and non-structural proteins P2 (2A, 2B, and 2C) and P3 (3A, 3B, 3C, and 3D) [2,7,8,9].

The prototype AiV-1 strain A846/88 was first isolated in 1989 in Aichi prefecture, Japan, from a fecal sample of a patient with oyster-associated acute gastroenteritis [4]. AiV-1 has been divided into two genetically distinct genotypes (A and B) on the basis of nucleotide sequences of the 3C and 3D (3CD) junction region [9,10]. An AiV-1 strain classified into a distinct genetic cluster was identified in France and proposed to represent third genotype of AiV-1, genotype C [11].

AiV-1 has been detected in human fecal samples in Asian [12,13,14,15], European [11,16,17,18,19,20], South American [18], and African continents [21,22], suggesting their worldwide distribution. In addition, seroprevalence studies performed in Japan [23], Germany [18], France [24], Spain [25], and Tunisia [26] demonstrated a high prevalence of AiV-1 antibodies in adults (80%–99%), indicating widespread human exposure. In contrast, a number of clinical investigations demonstrated a low incidence of AiV-1 infection in patients with either sporadic or epidemic gastroenteritis [11,12,13,14,15,16,17,18,19,20,21,22]. It has been reported that clinical signs and symptoms of AiV-1 infection typically include diarrhea, abdominal pain, nausea, vomiting, and fever [4,27]. However, recent clinical studies have shown that AiV-1 is usually present together with other enteric viruses in fecal samples of gastroenteritis patients [11,20]. These findings suggest: (1) AiV-1 might be circulating without causing any symptoms; (2) AiV-1 could be responsible for a portion of subclinical gastroenteritis infections requiring no medical attention; and/or (3) AiV-1 may contribute to mixed viral infections leading to enteric disease.

Recently, it has been revealed that AiV-1 is frequently detected in various types of environmental samples, such as sewage, river water, groundwater, and shellfish, suggesting that this virus is an emerging viral pathogen associated with environmental contamination and potentially with water and food borne infections.

## 2. Detection Methods

AiV-1 was first identified as a cytopathic virus in BS-C-1 cells [4]. Since then, various methods have been used to identify AiV-1 in clinical stool specimens, such as virus isolation using BS-C-1 or Vero cells [4,14], the enzyme-linked immunosorbent assay (ELISA) [23], and conventional reverse transcription (RT)-polymerase chain reaction (PCR) [10,13,28]. Isolation of AiV-1 by cell culture is time-consuming (about 4 to 6 weeks) [23], and both virus isolation and ELISA are less sensitive than conventional RT-PCR [10]. The conventional RT-PCR assay is a widely used method for AiV-1 identification because of its high sensitivity and applicability for further genetic analysis to determine genotypes [10]. In both clinical and environmental studies, genotype of AiV-1 has been determined based on nucleotide sequence analysis of the PCR products of 3CD junction or the capsid region [9,11,12,13,15,16,18,19,22,29,30,31,32]. The 3CD junction region is conserved and suitable for the detection of a wider range of AiV-1 genotypes, but the sequence data of this region does not seem to provide sufficient sequence diversity for subtyping [33]. Meanwhile, the capsid region (VP1), coding for structural proteins that express the antigenicity of the virus, is more genetically diverse and particularly suitable for distinguishing subtypes of AiV-1 [33]. However, no phylogenetic evidence of recombination has been obtained between AiV-1 genotypes A and B in any part of the genome [33], which suggests that 3CD- and capsid-based genotyping can result in the same genotype. Nested PCR assays targeting the 3C, VP1, and VP3 regions have also been developed [18,32] (see Figure 2) and used for the detection of Aichivirus A in water [32]. Pham *et al.* reported a multiplex semi-nested PCR assay using genotype-specific primers that target VP0-VP3 region for differentiation of genotypes A and B [28].

Real-time quantitative PCR (qPCR) allows rapid and quantitative detection of viral genomes. It even has many other advantages over conventional PCR, including lower risk of contamination, better specificity, and capability of multiplex reaction using multiple probes labeled with different reporter dyes. RT-qPCR assays for AiV-1 targeting the highly conserved 5' untranslated region (5'-UTR), a common target region for broadly reactive assay for picornaviruses, have been developed and used for determination of viral RNA load in fecal samples [30,34]. More recently, Kitajima *et al.* developed an RT-qPCR system targeting VP0 region (see Figure 2), which is able to quantify AiV-1 and differentiate between genotypes A and B [35]. This system consists of two assays, an AiV-universal assay utilizing a universal primer pair, and a universal probe and a duplex genotype-specific assay utilizing the same primer pair and two genotype-specific probes. A side-by-side comparison of cycle threshold (*C_T_*) values between one of the 5'-UTR qPCR assays [30] and the VP0 qPCR system [35] in detecting cDNA of genotype A and B strains showed that *C_T_* values obtained from the two qPCR systems were comparable for both genotype A and B strains [35]. The design of the VP0 qPCR system has an advantage over the 5'-UTR qPCR, in that a single pair of primers is used not only to simplify the assay but also to enable simultaneous detection of two genotypes with a similar PCR efficiency [35]. The VP0 RT-qPCR system will be an efficient tool for routine diagnosis of AiV-1 in clinical stool specimens as well as in environmental samples, because of its ability for both quantification and genotyping of AiV-1 [35]. Molecular characterization of AiV-1 in environmental samples requires cloning of the PCR products, since usually multiple AiV-1 strains exist in an environmental sample [31]; however, these procedures are labor intensive, time consuming, and requiring expensive reagents and equipment. The VP0 RT-qPCR system, which allows rapid, sensitive, and specific detection and can be used for quantitative analysis as well as for differentiation of genotypes using multiplex qPCR format, would contribute to facilitate further investigation on the prevalence and genotype distribution of AiV-1 in human populations as well as in the environment.

Environmental detection of enteric viruses with PCR can be affected by inhibitors in environmental samples. In order to assess the RT-qPCR efficiency for the detection of AiV-1 in environmental samples, a primer sharing control (PSC) RNA for AiV-1 was developed [36]. PSC RNA is an artificial RNA that has same sequence as the target viral gene fraction except for the TaqMan probe hybridization site [37]. Therefore, both target viral RNA and PSC RNA are expected to be reverse transcribed and amplified with the same efficiency in the same reaction tube as the target gene even in cases where RT-qPCR inhibition occurs due to the presence of inhibitors present in environmental samples [37]. The AiV-PSC-RNA developed by Hata *et al.* is compatible with the VP0 RT-qPCR system [35]; the PSC has 108 nucleotides of a sequence identical to the partial sequence (nucleotide location 1882–1989) of the prototype AiV-1 strain A846/88 (GenBank accession number AB040749) except for the sequence AiV-specific probe hybridizes, which was replaced with a foreign genome sequence (*i.e.*, complementary sequence of the murine norovirus-specific TaqMan probe MK-MNV-TP) [36].

## 3. Occurrence in the Environment

Table 1 summarizes recent reports on the occurrence of AiV-1 in the environment. AiV-1 excreted with human feces may contaminate surface waters directly or after discharge of treated or untreated sewage [31,32,36,38]. Fecal-oral AiV-1 transmission through contaminated food or water is indicated by AiV-1 detection in fecal samples of infected individuals as well as in raw and treated sewage [31,32,39], surface water [31,32,36,38], and shellfish [17,39,40]. Kitajima *et al.* and Lodder *et al.* reported the detection of both genotypes A and B in environmental samples in Japan and in the Netherlands, respectively [23,31,32], while the other environmental studies detected either genotype A or B. Genotype C has not been detected from environmental samples.

### 3.1. Sewage

The presence of viruses in sewage reflects the actual prevalence of the viruses in a community [41]. Kitajima *et al.* and Yamashita *et al.* reported that AiV-1 RNA was detected in 100% (12/12) and 66.2% (137/207), respectively, of raw sewage samples collected in Japan [31,42]. In the Netherlands, AiV-1 RNA was detected in 100% (16/16) of raw sewage samples by nested RT-PCR assays [32]. Sdiri-Lourizi *et al.* and Di Martino *et al.* also identified AiV-1 RNA in 15 of 250 (6%) raw sewage samples in Tunisia and 6 of 48 (12.5%) raw sewage samples in Italy, respectively [39,43]; the positive rate was far lower than that reported by Kitajima *et al.* [31] and Lodder *et al.* [32]. Sdiri-Lourizi *et al.* [39] and Di Martino *et al.* [43] probably underestimated the positive rate because they did not perform the second PCR that substantially increases detection sensitivity.

**Table 1:** Detection of Aichi virus 1 (AiV-1) in water and shellfish.

Sample		Detection Method	Positive Rate	Genotype	Concentration (copies/L)	Country	Reference
Water	Raw sewage	Nested PCR-cloning-sequencing	100% (12/12)	A + B	ND	Japan	[31]
		PCR-direct sequencing	8% (10/125)	A	ND	Tunisia	[39]
		qPCR	100% (12/12)	A	1.4 × 10^5^ to 2.2 × 10^7^	Japan	[35]
		Nested PCR-cloning-sequencing	100% (16/16)	A + B	ND	Netherlands	[32]
		PCR-direct sequencing	12.5% (6/48)	B	ND	Italy	[43]
		qPCR	100% (24/24)	AiV-1	1.2 × 10^4^ to 4.0 × 10^6^	US	[44]
		Viral metagenomics	50% (2/4)	AiV-1	ND	Spain, US	[45]
		Viral metagenomics	75% (3/4)	AiV-1	ND	Nepal, Thailand, US	[46]
		PCR-cloning-sequencing	66.2% (137/207)	A	ND	Japan	[42]
	Treated sewage	Nested PCR-cloning-sequencing	92% (11/12)	A	ND	Japan	[31]
		PCR-direct sequencing	4% (4/125)	A	ND	Tunisia	[39]
		qPCR	92% (11/12)	A	Up to 1.8 × 10^4^	Japan	[35]
		qPCR	100% (24/24)	AiV-1	2.0 × 10^3^ to 4.0 × 10^5^	US	[44]
		qPCR	61% (61/100)	AiV-1	Up to 10^3^	France	[47]
	Reclaimed water	Viral metagenomics	50% (1/2)	AiV-1	ND	US	[48]
	River water	PCR-direct sequencing	45% (5/11)	B	ND	Venezuela	[38]
		Nested PCR-cloning-sequencing	60% (36/60)	A + B	ND	Japan	[31]
		Nested PCR-cloning-sequencing	85% (12/14)	A + B	ND	Netherlands	[32]
		qPCR	100% (29/29)	AiV-1	8.6 × 10^2^ to 2.0 × 10^4^	Japan	[36]
		qPCR	11% (20/175)	ND	Up to 10^2^	France	[47]
Biosolids		Viral metagenomics	100% (1/1)	ND	ND	US	[49]
		Viral metagenomics	25% (3/12)	ND	ND	US	[50]
Shellfish	Clam	PCR-direct sequencing	33% (19/57)	A	ND	Japan	[40]
	Oyster	Nested PCR-direct sequencing	8% (5/62)	A	ND	France	[17]
	Mussel, clam, cockle	Nested PCR-hybridization	0% (0/41)	ND	ND	Spain	[51]
	Shellfish	PCR-direct sequencing	6.6% (4/60)	A	ND	Tunisia	[39]
	Oyster, clam, cockle	qPCR	0% (0/77)	ND	ND	Morocco	[52]

ND, not determined.

Kitajima *et al.* quantitatively detected AiV-1 RNA in raw sewage in Japan by RT-qPCR and reported its concentration ranging from 1.4 × 10^5^ to 2.2 × 10^7^ copies/L, which was the first report quantifying AiV-1 RNA in water [35]. Subsequently, the concentration of AiV-1 RNA in sewage in Arizona, USA was investigated using the VP0 RT-qPCR system [44]. In this study, AiV-1 was found in greater abundance without clear seasonality, and showed a lower reduction during wastewater treatment (0.94 ± 0.33 and 0.99 ± 0.12 log_10_ reduction by the whole wastewater treatment processes using activated sludge and trickling filter, respectively) than other human enteric viruses, such as noroviruses, sapoviruses, enteroviruses [44]. The results demonstrated the difficulty of achieving substantial physical removal of AiV-1 by the conventional wastewater treatment. Viruses discharged from wastewater treatment plants (WWTPs) can pose a risk of infection via various routes such as recreational water contact or virus accumulation in shellfish. WWTPs are one of the most effective modes of controlling circulation of viruses among human populations and aquatic environments; the more efficient removal of viruses from WWTP effluents before it is discharged into aquatic environments will directly contribute to reduction of viruses in the environment. Since recent studies demonstrated that AiV-1 showed relatively high abundance in both raw and treated wastewater [35,44], it could potentially be used as a conservative indicator of wastewater treatment system performance with respect to virus occurrence and removal.

### 3.2. River Water

Viral contamination of river water is of etiological significance, because river water is an important source of drinking water production for many regions of the world and often used for recreational purposes, such as swimming and bathing. AiV-1 was detected in 5 out of 11 (45%) sewage-polluted river water samples in Venezuela using the 3CD nested RT-PCR with a modified nested primer set (AiF2-AiR2b), which was the first study reporting the identification of AiV-1 in environmental water [38]. AiV-1 genotype identified in this study by direct sequencing of RT-PCR products was only genotype B. The detection and molecular characterization of AiV-1 in river water was subsequently reported from Japan, which identified AiV-1 in 36 out of 60 (60%) river water samples [31]. In this study, a total of 135 AiV-1 strains were identified from the river water by nucleotide sequencing of cloned nested RT-PCR products, of which only three clones obtained from one river sample were classified into genotype B and the rest of the strains were genotype A [31]. Interestingly, this study demonstrated more frequent detection of AiV-1 than human noroviruses and sapoviruses in the same set of river water samples [31].

### 3.3. Groundwater

One of our previous studies assessed the occurrence and removal of viruses at full-scale managed aquifer recharge (MAR) systems in three different regions of USA [53]. The concentration of AiV-1 in groundwater was up to 1.52 × 10^4^ copies/L. We also found that pepper mild mottle virus (PMMoV), a plant virus, was more abundant in groundwater than AiV-1 or any other enteric viruses tested. Since this is the only study investigating the occurrence of AiV-1 in groundwater so far, the behavior of AiV-1 in soil aquifer has not been well understood and warrants further investigation.

### 3.4. Shellfish

AiV-1 RNA was detected in 19 out of 57 (33%) packages of Japanese clams by RT-PCR, and only genotype A strains with over 95% nucleotide homology were identified by direct sequencing of the RT-PCR products [40]. Le Guyader *et al.* reported that AiV-1 genotype A was detected in 5 out of 62 (8%) oyster samples in France [17]. Sdiri-Loulizi *et al.* also reported that AiV-1 genotype A was detected in 4 out of 60 (6.6%) shellfish in Tunisia [39]. In contrast, Vilariño *et al.* and Bonabbes *et al.* reported that AiV-1 was not detected in shellfish from Spain and Morocco, respectively [51,52].

### 3.5. Persistence

AiV-1 RNA has been detected by PCR-based methods, but no previous study confirmed whether the viral RNA was derived from infectious particles. Nevertheless, these results indicate a potential risk of viral infection when treated wastewater is discharged into recreational areas or used to produce reclaimed water. Therefore, our future efforts should focus on developing methodologies for eliminating AiV-1 from these aquatic environments. Fortunately, AiV-1 can be easily propagated and assayed with routine cell culture system using Vero cells [23], which allows us to evaluate the effectiveness of disinfectants such as chlorine, UV, ozone in inactivating AiV-1.

It was suggested that AiV-1 could be readily inactivated within a day by dairy manure-based composting conditions mandated by the U.S. Environmental Protection Agency for Class A biosolids [54]. A few previous studies reported that AiV-1 was completely resistant to hydrostatic pressure treatments of up to 800 MPa, exhibiting generally higher resistance than other enteric viruses and surrogate viruses, such as coxsackieviruses, human parechovirus, feline calicivirus, murine norovirus, porcine enteric calicivirus, and Tulane virus [55,56,57].

AiV-1 was reported to be stable under a wide range of pH (2 to 10) and resistant (<0.5 log_10_ inactivation) against alcohol treatments with up to 90% isopropanol or ethanol for up to 5 min; in contrast, heat treatment at 56°C for 20 min readily inactivated AiV-1 (>4 log_10_ inactivation) [57]. AiV-1 on stainless steel disks seems to be insensitive to chlorine treatment (1.3 log_10_ inactivation by 1000 ppm for 5 min) [57].

## 4. Conclusion

AiV-1 has been proposed as a causative agent of human gastroenteritis potentially transmitted by fecal-oral routes through contaminated food or water; however, the epidemiology and etiological role of AiV-1 is, to a large extent, unknown. Recent environmental studies revealed that this virus could be detected in higher frequency and greater abundance than other human enteric viruses [31,36,44]. These findings suggest that AiV-1 could potentially be an appropriate indicator of viral contamination in the environment because of their high prevalence in water environments as well as structural and genetic similarity with some of other important enteric viruses. AiV-1 is a small round virus possessing a single-stranded RNA genome and is a member of the family *Picornaviridae* that includes enteroviruses and hepatitis A virus; these two viruses are listed in the latest U.S. Environmental Protection Agency’s Contaminant Candidate List (CCL3), which identifies emerging contaminants of aquatic environments that may pose a public health risk [58]. Further studies on the occurrence, behavior, and genotype distribution of AiV-1 in the environment, even in combination with clinical studies, of many regions are needed for a better understanding of its epidemiology, temporal and geographical distribution, environmental stability, and potential health risks to humans.
